# Glutamine deficiency promotes recurrence and metastasis in colorectal cancer through enhancing epithelial–mesenchymal transition

**DOI:** 10.1186/s12967-022-03523-3

**Published:** 2022-07-22

**Authors:** Hongyan Sun, Chuan Zhang, Yang Zheng, Chenlu Liu, Xue Wang, Xianling Cong

**Affiliations:** 1 Department of Biobank, China-Japan Union Hospital of Jilin University, Changchun, China; 2 Department of Pediatric Surgery, The First Hospital of Jilin University, Changchun, China; 3 Department of Dermatology, China-Japan Union Hospital of Jilin University, Changchun, China; 4 Health Promotion and Physical Examination Center, China-Japan Union Hospital of Jilin University, Changchun, China; 5 Department of Biobank, China-Japan Union Hospital of Jilin University, Changchun, China

## Abstract

**Background:**

Glutamine is the most abundant amino acid in the body and plays a vital role in colorectal cancer (CRC) cell metabolism. However, limited studies have investigated the clinical and prognostic significance of preoperative serum glutamine levels in patients with colorectal cancer, and the underlying mechanism has not been explored.

**Methods:**

A total of 121 newly diagnosed CRC patients between 2012 and 2016 were enrolled in this study. Serum glutamine levels were detected, and their associations with clinicopathological characteristics, systemic inflammation markers, carcinoembryonic antigen (CEA) and prognosis were analysed. In addition, the effect of glutamine depletion on recurrence and metastasis was examined in SW480 and DLD1 human CRC cell lines, and epithelial–mesenchymal transition (EMT)-related markers were detected to reveal the possible mechanism.

**Results:**

A decreased preoperative serum level of glutamine was associated with a higher T-class and lymph node metastasis (*P* < 0.05). A higher serum level of glutamine correlated with a lower CEA level (*r* = − 0.25, *P* = 0.02). Low glutamine levels were correlated with shorter overall survival (OS) and disease-free survival (DFS). Multivariate Cox regression analysis showed that serum glutamine was an independent prognostic factor for DFS (*P* = 0.018), and a nomogram predicting the probability of 1-, 3- and 5-year DFS after radical surgery was built. In addition, glutamine deficiency promoted the migration and invasion of CRC cells. E-cadherin, a vital marker of EMT, was decreased, and EMT transcription factors, including zeb1and zeb2, were upregulated in this process.

**Conclusions:**

This study elucidated that preoperative serum glutamine is an independent prognostic biomarker to predict CRC progression and suggested that glutamine deprivation might promote migration and invasion in CRC cells by inducing the EMT process.

**Supplementary Information:**

The online version contains supplementary material available at 10.1186/s12967-022-03523-3.

## Introduction

Colorectal cancer (CRC) is the fourth most common malignant tumour worldwide and the second leading cause of cancer death [1]. Despite recent advancements in surgical resection and adjuvant therapies in the treatment of CRC, a number of CRC patients, especially advanced CRC patients, still develop local recurrence or distant metastasis, which ultimately leads to death. The 5-year survival rate was only slightly greater than 10% in patients with stage IV disease [2].

Reprogramming energy metabolism is an emerging hallmark of cancer, and many cancer cells rely on glutamine for growth and division [3]. Glutamine serves as an important source of carbon for the synthesis of nucleotides and fatty acids and a source of nitrogen directly for the biosynthesis of purines and pyrimidines in these cancer cells [4].

In colorectal cancer, only two recent studies investigated the clinical significance of glutamine levels in patients with colorectal cancer and reported that low serum glutamine was associated with elevated systemic inflammation and poorer prognosis [5, 6]. The results of these studies need further verification in in vivo and in vitro experiments, and the underlying mechanism has not been elucidated.

Epithelial–mesenchymal transition (EMT) refers to the transformation of epithelial cells into mesenchymal cells, which allows cancer cells to exhibit increased cell motility and acquire invasive potential. EMT is a critical process for the initiation of the metastatic cascade in colorectal cancer [7]. E-cadherin is a type I transmembrane glycoprotein that mainly regulates cell–cell adhesions and plays a vital role in maintaining the normal structure of epithelial tissues. Decreased expression of E-cadherin is an important hallmark of EMT. Upregulation of EMT transcription factors, such as Snail, Twist, and Zeb, promotes EMT by inhibiting the expression of E-cadherin and inducing the expression of mesenchymal markers, such as fibronectin, vimentin and N-cadherin [8–11]. In addition, the EMT process can also be modulated by some signalling pathways, such as the WNT/β-catenin and TGF-β signalling pathways [12, 13]. At present, how glutamine deficiency affects EMT and metastasis and the regulation of EMT-TFs and signalling pathways in colorectal cancer have not been explored.

In this study, we evaluated the clinical significance of glutamine in colorectal cancer and found that low preoperative glutamine levels were associated with poor prognosis. The underlying mechanism was further explored, and glutamine deficiency might promote metastasis by enhancing EMT.

## Materials and methods

### Patients

A total of 121 newly diagnosed CRC patients in the China-Japan Union Hospital of Jilin University between January 2012 and June 2016 were enrolled in this study. All patients signed informed consent forms to participate in the study before radical resection of colorectal cancer. Blood samples were collected and stored in the biobank of China-Japan Union Hospital of Jilin University. The clinicopathological and systemic inflammation information for 121 CRC cases was collected from each patient’s medical records.

Follow-up assessments of CRC patients were scheduled every 3 months for the first 2 years after surgery, every 6 months for the next 3 years, and once a year thereafter. During routine follow-up, chest and abdominal computed tomography, tumour marker measurements, and endoscopy were performed every 6 months. Complete follow-up data were collected until June 2019 or death for all CRC patients. Disease-free survival (DFS) was calculated from the date of tumour resection until the detection of tumour recurrence or last observation, and overall survival (OS) was calculated from the date of resection to the date of death or last follow-up. This study was approved by the Ethical Committee of the China-Japan Union Hospital of Jilin University and performed according to the principles of the Declaration of Helsinki.

### Measurement of glutamine and CEA

A Glutamine Colorimetric Assay Kit (K556-100, BioVision) was used to measure serum glutamine levels. Blood was collected preoperatively, and none of the patients had received radiotherapy, chemotherapy or supplementation with glutamine before surgery. Serum was obtained by centrifugation and stored at − 80 °C until detection. A parallel sample well was set to eliminate the influence of the background signal, and each sample was tested twice. Glutamine levels were calculated from the standard curve.

Carcinoembryonic antigen (CEA) is a classic tumour biomarker for CRC, and the serum CEA test has been widely used to monitor the presence of primary CRC and recurrent CRC following radical resection in clinical practice. Radioimmunoassays were used to determine the CEA in serum at the Department of Nuclear Medicine in the China-Japan Union Hospital of Jilin University. The normal reference value was between 0.5 and 9.6 µg/L.

### Cell culture

The human colorectal cancer cell lines SW480 and DLD1 were obtained from American Type Culture Collection and cultured in our laboratory. Both cell lines were cultured in Dulbecco’s modified Eagle’s medium (DMEM; Gibco, Thermo Fisher Scientific, Massachusetts, MA) supplemented with 10% fetal bovine serum (Thermo Fisher Scientific) and 100 U/mL penicillin–streptomycin at 37 °C in a humidified atmosphere containing 5% CO_2_. Glutamine deficiency experiments were performed in glutamine-free DMEM (Gibco11960044), and different volumes of 200 mM glutamine storage solution were added to achieve the required concentration.

### Migration and invasion assays

For the wound healing assay, five straight lines were drawn on the back of the 6-well plates, and SW480 and DLD1 cells were seeded in 6-well plates in complete culture medium and cultured to almost 100% confluence the next day. The cell layer was carefully wounded using a 200 μL sterile tip perpendicular to the straight lines and washed twice with PBS. Then, the cells were cultured in serum-free medium and treated with different concentrations of glutamine. Wound margins were photographed at 0 and 24 h in 5 randomly selected microscopic regions. The migration rate was calculated to determine the migration ability of cells treated with normal or low concentrations of glutamine.

Cell migration and invasion assays were performed by using transwell chambers (pore size 8.0 µm; Costar, Corning, Switzerland) without or with Matrigel (Cat. No. 354235, BD). SW480 and DLD1 cells were cultured for 24 h in 4 mM, 1 mM or 0.5 mM glutamine DMEM without serum before seeding in transwell chambers. Then, 100 μL serum-free medium containing 5 × 10^4^ cells and different concentrations of glutamine were added to each upper chamber, and 600 µL medium containing 15% FBS was added to each lower chamber. After incubation for 24–48 h, cells that migrated/invaded through the insert membrane were fixed with cold 100% methanol for 20 min and then stained with 0.1% crystal violet solution for 20 min, rinsed with PBS, and the noninvasive/nonmigratory cells in the upper surface of the membrane were removed with cotton swabs. Finally, images were taken at 20× magnification with an Olympus IX-53 microscope. Four fields within each transwell chamber were chosen to count the number of migrating or invading cells.

### RNA extraction and qPCR

Total RNA was extracted from cells with TRIzol™ reagent (Thermo Fisher Scientific). RNA (500 ng) was converted into cDNA using the PrimeScript™ RT reagent Kit with gDNA Eraser (Perfect Real Time) (TaKaRa, RR047A). mRNA expression levels were quantified by qPCR using TB GreenPremix Ex TaqTM II (Tli RNaseH Plus) (TaKaRa, RR820A) in triplicate, and the primers are listed in Additional file 1: Table S1. The reaction was performed with the ABI StepOnePlus Real-Time PCR System using a two-step amplification procedure. Relative target gene expression was determined by comparing the average threshold cycles with that of the housekeeping gene GAPDH by the 2^−ΔΔCt^ method.

### Immunostaining

SW480 cells were seeded on slides in a 24-well plate at an appropriate density overnight and then cultured with low serum culture medium with 4 mM or 1 mM glutamine. After 48 h, the cells were fixed in cold 4% paraformaldehyde for 20 min and then blocked by incubation with 10% normal goat serum for 30 min at room temperature to eliminate nonspecific staining. The cells were then incubated overnight at 4 °C with anti-E-cadherin diluted at 1:200 (#3195S; CST) and incubated with Cy3-conjugated secondary antibodies (abs20024; Absin, China) for 1 h at 37 °C and DAPI for 5 min at room temperature. After the cells were washed with PBST, Olympus IX-53 fluorescence microscopy was used to observe the expression of target proteins in different groups of cells.

### Western blot

Cells were lysed in RIPA Lysis Buffer (P0013B; Beyotime, China) supplemented with protease inhibitors, and protein concentrations were measured using the BCA Protein Assay Kit (P0010; Beyotime, China). Protein samples (50–100 μg) were separated by SDS-PAGE and transferred to polyvinylidene difluoride membranes (Millipore). After blocking with 5% fat-free dry milk in TBST, the blots were incubated overnight at 4 °C with primary antibodies, including anti-E-cadherin (24E10, 1:1000; CST), anti-Snail (C15D3, 1:1000; CST), anti-Slug (C19G7, 1:1000; CST), anti-Zeb1 (E2G6Y, 1:1000; CST), anti-Zeb2 (14026-1-AP, 1:1000; proteintech). Anti-GAPDH (D16H11, 1:1000; CST) was used as an internal control for the total proteins. Then, the membranes were incubated with IRDye800CW secondary antibodies (LI-COR) and detected using an Odyssey infrared imaging system (LI-COR).

### Statistical analysis

Statistical analyses were performed using IBM SPSS Statistics for Windows, version 25.0 (IBM Corporation, Armonk, NY, USA). Normally distributed continuous variables were presented as the mean ± SD (standard deviation). Correlations between serum glutamine levels and clinicopathological characteristics or systemic inflammation in CRC patients were analysed by independent samples t test or one-way ANOVA. Receiver operating characteristic (ROC) analysis was used to determine the optimal serum glutamine cut-off value in detecting patients who survived during follow-up. Survival curves were calculated using the Kaplan–Meier method, and intergroup differences were compared using the log-rank test. A Cox proportional hazards regression model was used to assess the independent prognostic significance of glutamine. A nomogram was drawn with R version 3.6.1 (R Foundation for Statistical Computing, Vienna, Austria). All of the tests were two-tailed, and *P* < 0.05 was considered as statistically significant.

## Results

### Associations of serum glutamine levels with the clinicopathological characteristics of CRC patients

The correlations between serum glutamine levels and clinicopathological features in 121 CRC patients are shown in Table 1. A higher T-class was associated with a decreased serum level of glutamine (*P* = 0.007), and lymph node metastasis was also associated with a decreased serum level of glutamine (*P* = 0.037). Moreover, the serum level of glutamine was decreased in patients with postoperative recurrence or metastasis (*P* = 0.008).

**Table 1:** Associations of serum glutamine level with clinicopathological characteristics of CRC patients

Variable	No. of cases	Glutamine (μM)		*P* values
		Mean	SD	
Age (year)				0.147
≥ 65	56	593.52	418.88	
< 65	65	493.14	337.12	
Sex				0.860
Male	73	534.65	344.40	
Female	48	547.11	429.81	
BMI				0.278
< 18.5	12	383.02	343.87	
18.5–23.9	64	577.94	394.39	
24–26.9	28	480.44	378.68	
≥ 27	17	603.22	326.02	
Tumor size (cm)				0.503
≥ 5	68	519.12	373.75	
< 5	53	565.87	387.38	
Tumor location				0.161
Colon	61	491.58	349.99	
Rectum	60	588.41	403.25	
Tumor differentiation				0.563
Well and moderate	93	550.60	376.72	
Poor	28	503.05	390.74	
Depth of invasion				0.007*
Tis–T2	23	601.45	360.58	
T3	82	575.39	387.13	
T4	16	267.23	237.72	
Lymph node metastasis				0.037*
Yes	59	466.17	409.97	
No	62	609.47	335.31	
Venous permeation				0.161
Yes	55	486.62	416.73	
No	66	583.75	341.18	
Perineural invasion				0.94
Yes	27	534.73	362.30	
No	94	540.99	385.40	
CEA				
High	21	435.13	309.45	0.017*
Normal	60	636.65	329.39	
Survival status				0.259
Death	42	486.07	427.31	
Survival	79	568.05	350.03	
Metastasis/recurrence				0.008*
Yes	49	430.11	372.35	
No	72	614.11	367.43	

*BMI* body mass index, *CEA* carcinoembryonic antigen

*Statistically significant

### Relationship of serum glutamine levels and systemic inflammatory markers in CRC patients

Some haematologic parameters have been identified to evaluate the systemic inflammatory response, such as the neutrophil/lymphocyte ratio (NLR), derived neutrophil–lymphocyte ratio (dNLR), platelet/lymphocyte ratio (PLR), the combination of platelet count and neutrophil lymphocyte (COP–NLR), lymphocyte/monocyte ratio (LMR), and prognostic nutritional index (PNI). These indicators have been demonstrated to be associated with tumour progression and metastasis, so we analysed the relationship between serum glutamine levels and systemic inflammatory markers in CRC patients, and no association was found between serum glutamine levels and these indicators (Additional file 1: Table S2).

### Associations of serum glutamine levels with CEA levels in CRC patients

We further evaluated the correlation between serum glutamine levels and CEA levels in 81 CRC patients. The glutamine level was 636.65 ± 329.39 μM in the normal CEA group and 435.13 ± 309.45 μM in the high CEA group. The serum level of glutamine was decreased in patients with high CEA CRC (*P* = 0.017, Table 1). A higher serum level of glutamine correlated with a lower CEA level (r = − 0.25, *P* = 0.02, Additional file 1: Fig. S1).

### Prognostic value of serum glutamine levels in CRC patients

According to the ROC analysis in the present study, the optimal glutamine cut-off value based on the survival status was 392.83 μM, with an area under the curve of 0.646 (95% confidence interval (CI) 0.554–0.731; *P* < 0.05, Additional file 1: Fig. S2).

**Fig. 1:**
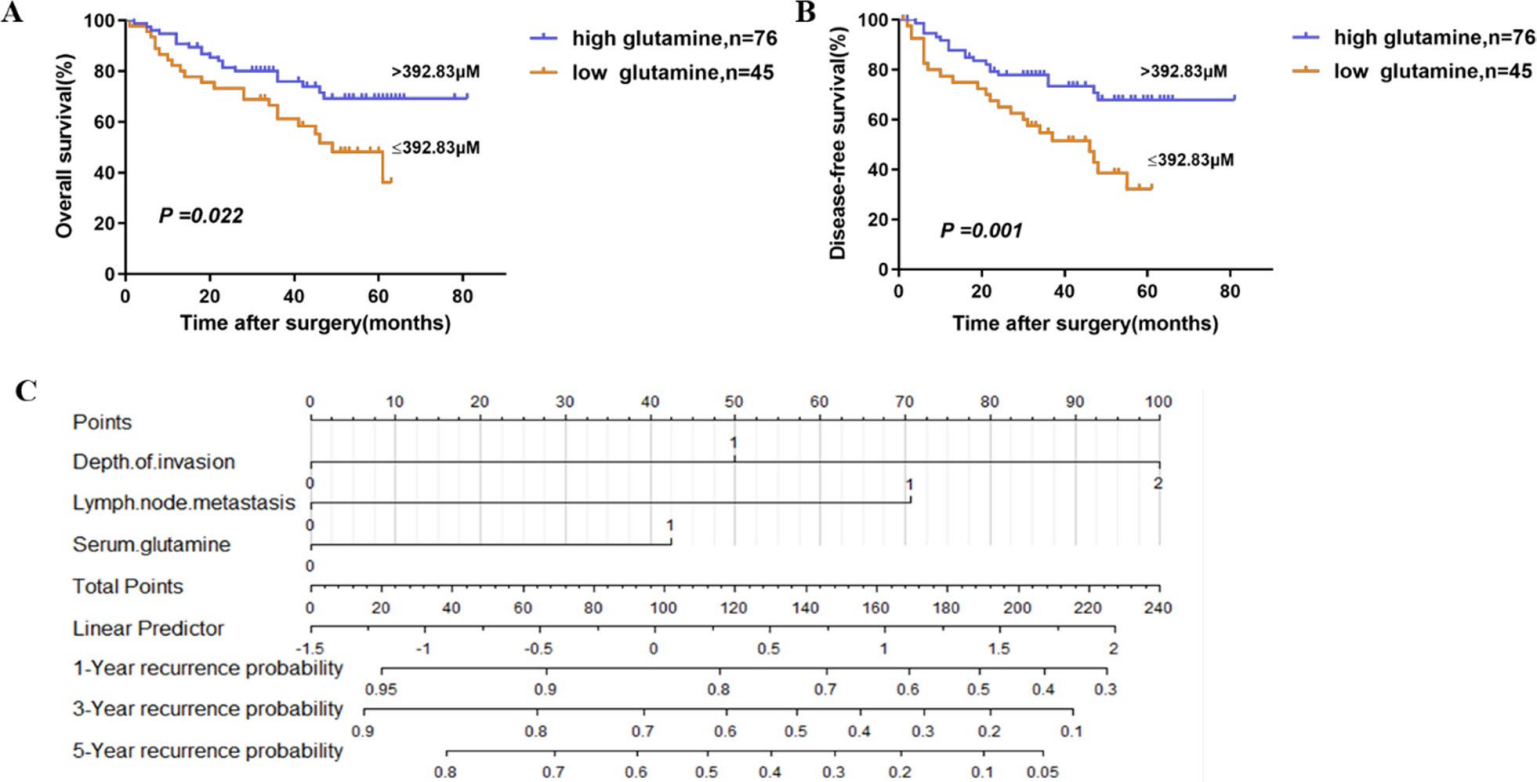
The prognostic significance of serum glutamine evaluated by Kaplan–Meier analysis. The OS (**A**) and DFS (**B**) of patients with low serum glutamine level were significantly shorter than that of patients with high level. **C** DFS nomogram based on the multivariate model. Depth of invasion: 0 (Tis–T2) vs. 1 (T3) vs. 2 (T4), Lymph node metastasis: 0 (no) vs. 1 (yes), serum glutamine: 0 (> 392.83 μM) vs. 1 (≤ 392.83 μM)

A Kaplan–Meier analysis of OS showed increased survival in patients with high glutamine levels compared to patients with low glutamine levels (log-rank, *P* = 0.022) (Fig. 1A). The mean OS was 63.58 months in the high glutamine level group, whereas it was only 42.68 months in the low glutamine level group. Moreover, a low glutamine level was correlated with shorter DFS (log-rank, P = 0.001). The mean DFS was 60.60 months for patients with high glutamine levels compared to 33.96 months for patients with low glutamine levels (Fig. 1B).

Univariate analysis showed that 8 factors, including BMI (*P* = 0.030), tumour differentiation (*P* = 0.013), depth of invasion (*P* = 0.002), lymph node metastasis (*P* = 0.029), venous permeation (*P* = 0.004), perineural invasion (*P* = 0.003), adjuvant therapy (*P* = 0.000) and serum glutamine (*P* = 0.025), were associated with OS. Seven factors showed a significant correlation with DFS (Table 2). These included tumour location (*P* = 0.049), tumour differentiation (*P* = 0.000), depth of invasion (*P* = 0.003), lymph node metastasis (*P* = 0.000), venous permeation (*P* = 0.000), perineural invasion (*P* = 0.002), and serum glutamine (*P* = 0.002). Multivariate analysis further confirmed that serum glutamine was an independent prognostic factor for DFS (*P* = 0.018) but not for OS (Table 3). The nomogram predicting the probability of 1-, 3- and 5-year DFS after radical surgery are shown in Fig. 1C.

**Table 2:** Univariate analysis of prognostic factors associated with survival and recurrence

Variables	OS		DFS	
	Hazard ratio (95% CI)	*P* value	Hazard ratio (95% CI)	*P* value
Age (year)				
≥ 65 versus < 65	1.513 (0.823, 2.782)	0.183	0.910 (0.516, 1.602)	0.743
Gender				
Male versus female	0.870 (0.462, 1.637)	0.666	1.000 (0.563, 1.776)	0.999
BMI		0.030*		0.833
18.5–23.9 vs. < 18.5	0.374 (0.160, 0.876)	0.024*	0.710 (0.272, 1.856)	0.485
24–26.9 vs. < 18.5	0.260 (0.093, 0.724)	0.010*	0.757 (0.269, 2.132)	0.598
≥ 27 vs. < 18.5	0.191 (0.049, 0.742)	0.017*	0.563 (0.162, 1.948)	0.364
Tumor size(cm)				
≥ 5 versus < 5	0.624 (0.328, 1.187)	0.151	1.260 (0.708, 2.242)	0.433
Tumor location				
Colon versus rectum	1.840 (0.987, 3.432)	0.055	1.785 (1.003, 3.179)	0.049*
Tumor differentiation				
Well and moderate versus poor	0.244 (0.131, 0.454)	0.013*	0.337 (0.186, 0.611)	0.000*
Depth of invasion		0.002*		0.003*
T3 versus Tis–T2	1.984 (0.693, 5.681)	0.202	5.902 (1.419, 24.556)	0.015*
T4 versus Tis–T2	5.706 (1.815, 17.946)	0.003*	12.019 (2.656, 54.385)	0.001*
Lymph node metastasis				
Yes versus no	2.021 (1.074, 3.805)	0.029*	3.381 (1.816, 6.293)	0.000*
Venous permeation				
Yes versus no	2.544 (1.352, 4.786)	0.004*	3.038 (1.680, 5.497)	0.000*
Perineural invasion				
Yes versus no	2.572 (1.377, 4.805)	0.003*	2.565 (1431, 4.598)	0.002*
Adjuvant therapy				
Yes versus no	0.285 (0.147, 0.552)	0.000*	0.569 (0.323, 1.004)	0.052
Serum glutamine				
≤ 392.83versus > 392.83	1.998 (1.090, 3.663)	0.025*	2.464 (1.402, 4.330)	0.002

*OS* overall survival, *DFS* disease-free survival, *95% CI* 95% confidence interval

*Statistically significant

**Table 3:** Multivariate cox regression analysis of prognostic factors associated with OS and DFS

Variables	Multivariate analysis	
	Hazard ratio (95% CI)	*P* value
OS		
Tumor differentiation		
Well and moderate versus poor	0.278 (0.147, 0.529)	0.000*
Perineural invasion		
Yes versus no	2.323 (1.228, 4.395)	0.010*
Adjuvant therapy		
Yes versus no	0.262 (0.133, 0.516)	0.000*
DFS		
Depth of invasion		
T3 versus Tis–T2	4.882 (1.166, 20.442)	0.03*
T4 versus Tis–T2	7.623 (1.665, 34.892)	0.009*
Lymph node metastasis		
Yes versus no	2.780 (1.484, 5.209)	0.001*
Serum glutamine		
≤ 392.83 versus > 392.83	2.021 (1.128, 3.622)	0.018*

*OS* overall survival, *DFS* disease-free survival

*Statistically significant

### Glutamine deprivation promotes migration and invasion in CRC cells by reducing E-cadherin

**Fig. 2:**
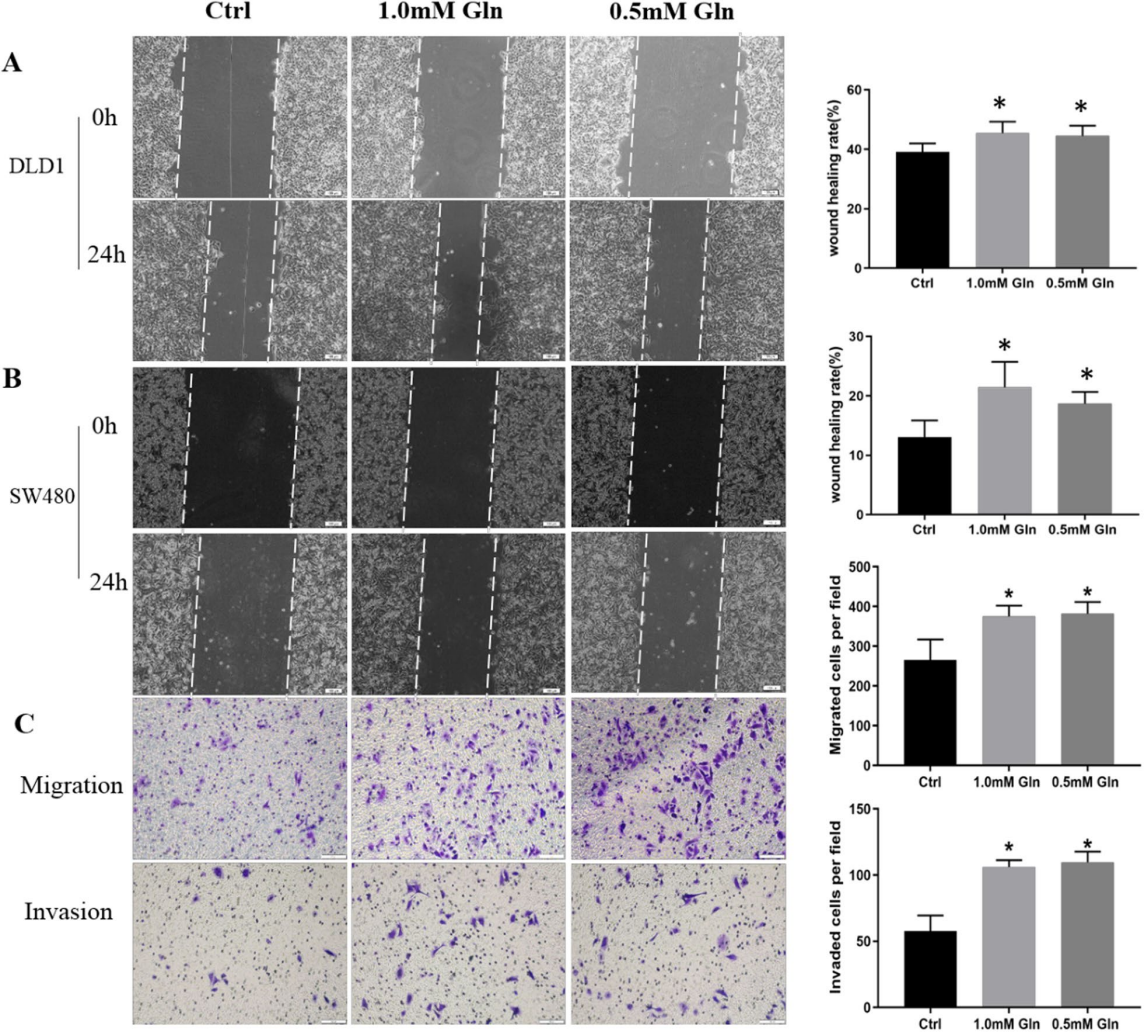
Glutamine deprivation promotes migration and invasion in CRC cells. **A** Glutamine depletion significantly increased the wound healing rate compared with normal glutamine concentration in DLD1 cells. **B** Glutamine depletion significantly increased the wound healing rate compared with normal glutamine concentration in SW480 cells at 24 h. **C** Glutamine depletion enhanced both the migration and invasion capacities of SW480 cells

Glutamine deficiency significantly increased the wound healing rate compared with the normal glutamine concentration in SW480 cells (1.0 mM group vs. normal group, 21.44 ± 4.3% vs. 13.07 ± 2.79%, *P* < 0.05; 0.5 mM group vs. normal group, 18.68 ± 1.97% vs. 13.07 ± 2.79%, *P* < 0.05) and DLD1 cells (1.0 mM group vs. normal group, 45.44 ± 3.85% vs. 39.07 ± 2.91%, *P* < 0.05; 0.5 mM group vs. normal group, 44.50 ± 3.41% vs. 39.07 ± 2.91%, *P* < 0.05) at 24 h (Fig. 2A, B). In addition, we found that limitation of glutamine enhanced both the migration (1.0 mM group vs. normal group, 375 ± 27 vs.265 ± 52, *P* < 0.05; 0.5 mM group vs. normal group, 382 ± 30 vs. 265 ± 52, *P* < 0.05) and invasion (1.0 mM group vs. normal group, 106 ± 5 vs. 57 ± 12, *P* < 0.05; 0.5 mM group vs. normal group, 110 ± 8 vs. 57 ± 12, *P* < 0.05) capacities of SW480 cells (Fig. 2C).

**Fig. 3:**
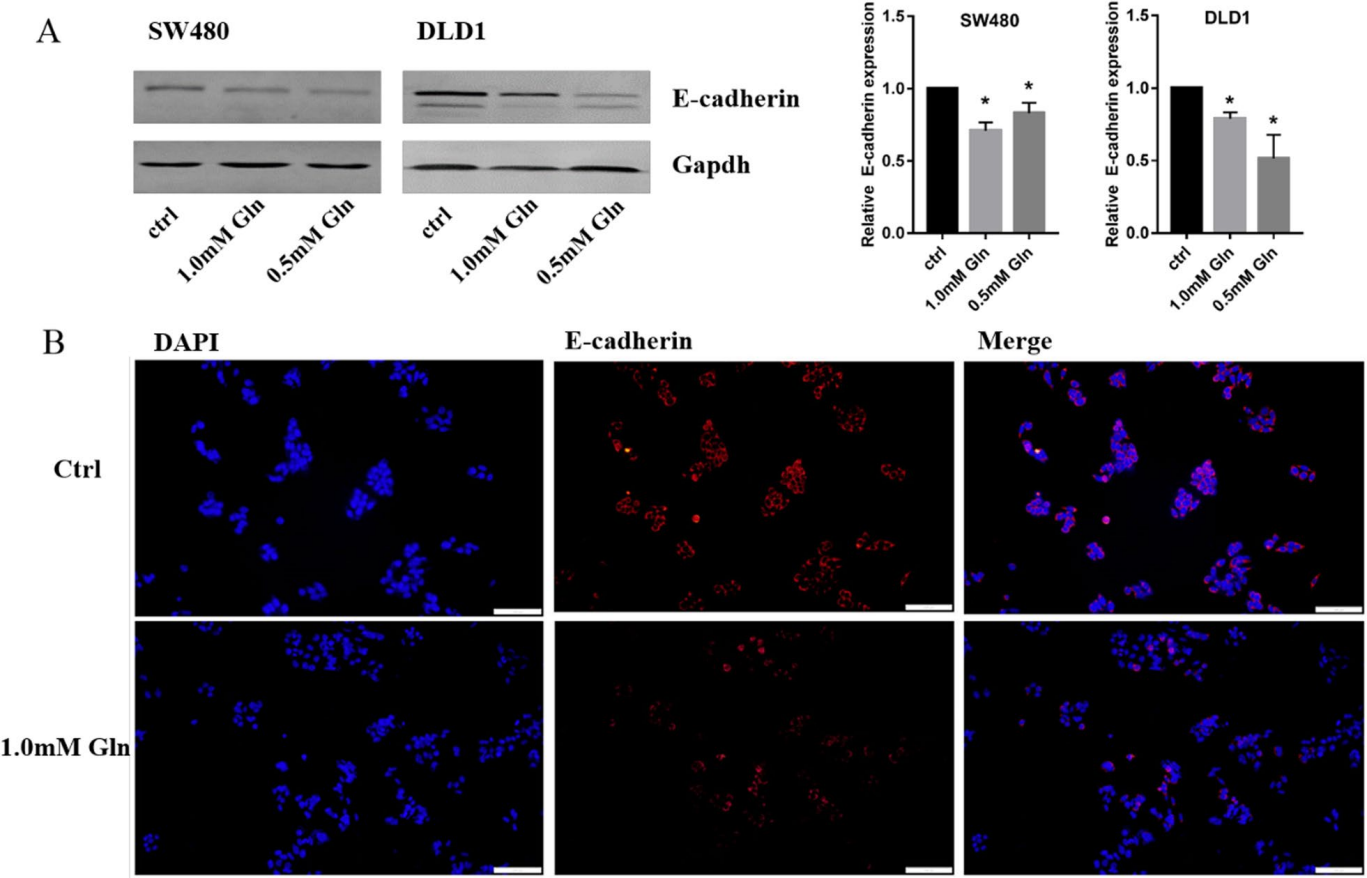
Glutamine depletion inhibited E-cadherin expression in CRC cells. **A** Western blot analysis showed that the protein levels in 1.0 mM and 0.5 mM glutamine groups decreased compared with normal glutamine groups in SW480 and DLD1 cells. The relative integrated density was 0.71 ± 0.04 in SW480 1.0 mM, 0.83 ± 0.05 in SW480 0.5 mM glutamine group; 0.79 ± 0.03 in DLD1 1.0 mM glutamine group, 0.52 ± 0.12 in DLD1 0.5 mM glutamine group, *P < 0.05; **B** immunostaining results showed that E-cadherin expression was decreased in 1.0 mM glutamine group compared with normal glutamine group in SW480 cells (×200)

E-cadherin is an important protein closely related to tumour invasion and metastasis. Downregulation of E-cadherin is a key feature of EMT. Glutamine deficiency inhibited E-cadherin expression in CRC cells. The protein levels of E-cadherin were evaluated by western blotting, and the results showed that the protein levels in the 1.0 mM and 0.5 mM glutamine groups decreased sequentially compared with those in the normal glutamine groups in SW480 and DLD1 cells (Fig. 3A). E-cadherin expression was also detected by immunostaining in SW480 cells in the normal glutamine group and 1.0 mM glutamine group, and the results showed the same tendency as the western blot results (Fig. 3B).

### Glutamine depletion affects the expression of transcription factors associated with EMT in colorectal cancer

**Fig. 4:**
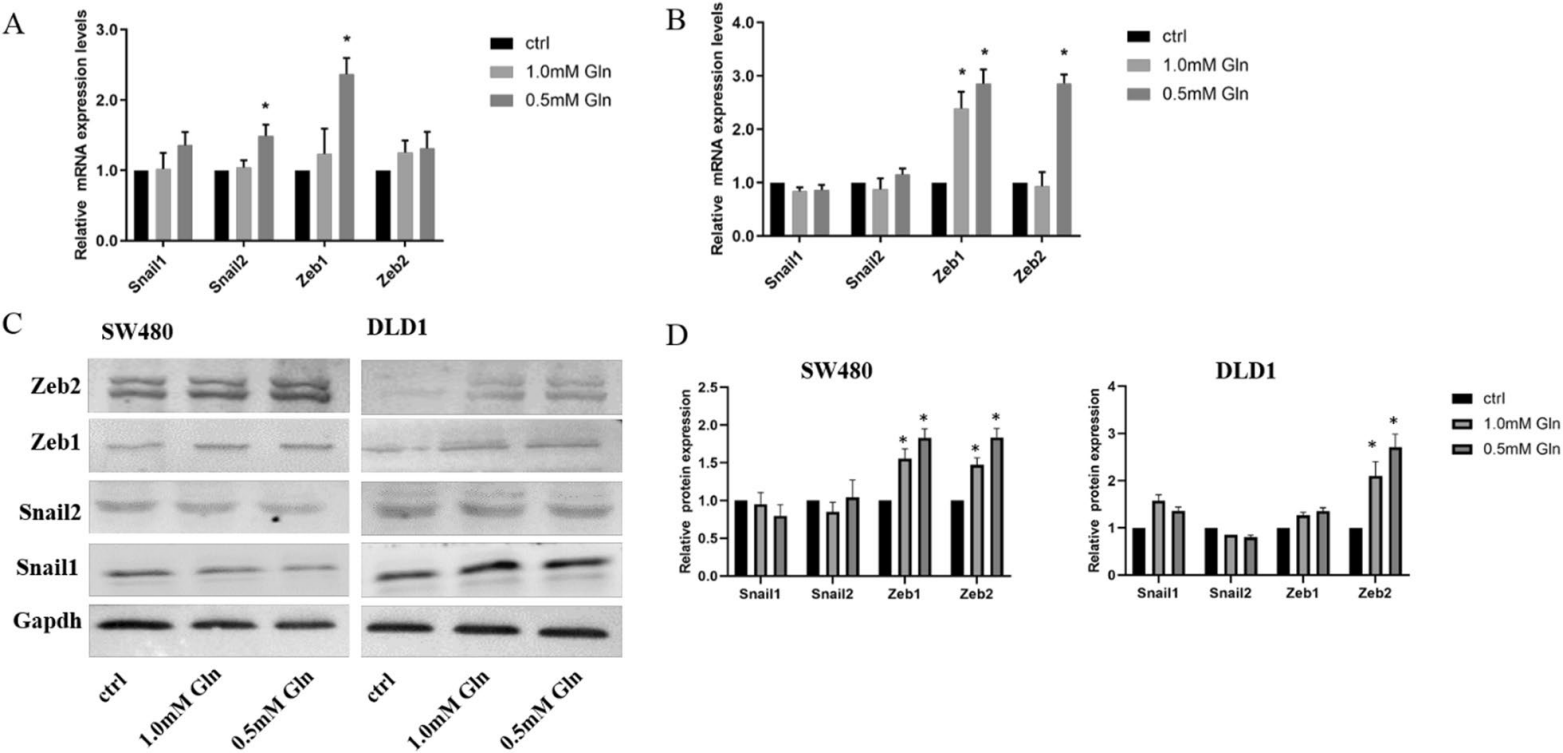
The different mRNA and protein expression of TFs in CRC cells treated with different glutamine concentration. **A** The mRNA expression of zeb1 and snail 2 were increased in 0.5mM glutamine defiency group in SW480 cells. **B** The results showed that the zeb1 mRNA expression in the 0.5 mM and 1.0 mM glutamine deficiency groups were significantly increased compared with the normal glutamine group in DLD1 cells. Zeb2 mRNA expression was also upregulated in the 0.5 mM glutamine deficiency group in DLD1 cells. **C** Protein expression of TFs in CRC cells treated with different glutamine concentration. **D** The results of western blot showed that zeb1 and Zeb2 in glutamine deficiency groups were significantly increased compared with the normal glutamine group in SW480 cells. Zeb2 in glutamine deficiency groups were significantly increased compared with the normal glutamine group in DLD1 cells. *P < 0.05, compared with control group

At present, how glutamine depletion affects migration and invasion has not been explored, despite the fact that EMT is an important step in tumour metastasis. We detected the expression of TFs in different glutamine concentration groups, including snail1, snail2, zeb1 and zeb2. The results of qPCR showed that the expression of zeb1 and snail2 was increased in the 0.5 mM glutamine deficiency group in SW480 cells (Fig. 4A). Zeb1 expression in the 0.5 mM and 1.0 mM glutamine deficiency groups was significantly increased compared with that in the normal glutamine group in DLD1 cells. Another transcription factor, zeb2, was also upregulated in the 0.5 mM glutamine deficiency group in DLD1 cells (Fig. 4B). The results of western blot showed that zeb1 and zeb2 in glutamine deficiency groups were significantly increased compared with the normal glutamine group in SW480 cells. Zeb2 in glutamine deficiency groups were also significantly increased compared with the normal glutamine group in DLD1 cells (Fig. 4C, D).

## Discussion

Metabolic reprogramming is a hallmark of cancer cells, in addition to the Warburg effect, many types of cancer cells are characterized by glutamine dependency [14, 15]. In most cancer cells, glutamine is deaminated by glutaminases to glutamate, and then glutamate is converted to α-ketoglutarate and enters the TCA cycle to produce ATP. In addition, the metabolic products from glutamine are utilized to synthesize macromolecules that promote tumour growth, including amino acids, fatty acids, purines and pyridines [16].

In colorectal cancer, increased glutamine metabolism is involved in cancer cell migration, invasion, and metastatic colonization [17]. In this study, we systematically evaluated the clinical significance of glutamine in colorectal cancer and found that decreased preoperative serum levels of glutamine were associated with higher T-class and lymph node metastasis. In addition, complete follow-up data were collected, and survival analysis was performed. The results showed that a low glutamine level was correlated with shorter overall survival (OS) and disease-free survival (DFS). Several studies have reported that serum glutamine levels in colorectal patients are lower than those in healthy controls [18–21]. Päivi Sirniö reported that low serum glutamine was associated with advanced disease stage and systemic inflammation, and low levels of serum glutamine were also associated with poor cancer-specific survival in univariate analysis [6]. Ling et al. further analysed the clinical significance of serum glutamine levels in patients with colorectal cancer and found that patients with low glutamine levels had elevated levels of proinflammatory cytokines, and pretreatment glutamine levels independently predicted OS and PFS in multivariate analysis in CRC patients [5], which was different from previous Päivi Sirniö’s study [6]. The prognostic factors influencing prognosis included in these two studies were quite different, and the cut-off value was 410 μΜ in Päivi Sirniö’s study [6], while it was 52 ng/μL (356 μΜ) with an area under the curve of 0.279 in Ling’s study. The cut-off value was determined by ROC curve, the area under the curve was less than 0.5, and the predictive value of the model was not very effective [5]. The optimal glutamine cut-off value was 392.83 μM with an area under the curve of 0.646 in this study, and the larger area under the curve than that in a previous study increased the predictive value. The results of multivariate Cox regression analysis using this cut-off value further showed that serum glutamine was an independent prognostic factor for DFS.

Our results also showed that a higher serum level of glutamine correlated with a lower CEA level (*r* = − 0.25, *P* = 0.02). Studies have shown that elevated serum levels of CEA correlate with the presence of primary CRC and recurrent CRC following radical resection. In terms of CRC diagnosis, CEA has been proven to be a sensitive marker for the early diagnosis of colorectal cancer, and many studies have combined CEA with other markers to improve the specificity of tumour diagnosis, which indicates that glutamine might be used in combination with CEA in the diagnosis of tumours in the future, not only in prognostic prediction [22, 23].

Prognostic indicators of colorectal cancer were considered as comprehensively as possible in this study. In addition to common clinicopathological features and inflammatory indicators, postoperative adjuvant therapy information was also collected during the follow-up and analysed in the survival analysis. Previous studies on the relationship between glutamine and prognosis did not consider the effect of this indicator. Multivariate analysis confirmed that adjuvant therapy was an independent prognostic factor for OS in this study. As a wider range of prognostic indicators were analysed in Cox regression analysis, the result of the Cox multivariate regression model was more credible in this study and shown intuitively by a nomogram.

In addition to glucose, glutamine is another major fuel for immune cells, including lymphocytes, neutrophils, and macrophages [24]. Studies have shown that the glutamine utilization rate is similar to or higher than the glucose utilization rate in immune cells under catabolic conditions, such as infection, postinjury or surgery, malnutrition, and high-intensity physical exercise [25, 26]. Our results and previous studies have shown that serum glutamine levels are decreased in colorectal cancer; however, limited research has investigated whether this decrease is associated with immune status. Sirniö et al. found that low serum glutamine levels were associated with indicators of systemic inflammation, including a high modified Glasgow Prognostic Score, high blood neutrophil/lymphocyte ratio and high serum levels of CRP, IL-6 and IL-8 [6]. Ling et al. found that low glutamine levels were associated with higher C-reactive protein levels, higher modified Glasgow prognostic scores, and higher IL-6 and IL-1β levels [5]. The association between serum glutamine levels and indicators of systemic inflammation was also evaluated in our study, and no association was found between serum glutamine levels and NLR, which was different from Sirniö’s study [6]. Other indicators, including the dNLR, PLR, COP–NLR, LMR and PNI, were first analysed for their relationship with glutamine levels in this study. These indicators did not show statistically significant correlations with glutamine, which might be due to the instability of the routine blood index of hospitalized CRC patients and the relatively small sample size.

Recently, glutamine supplementation has been reported to reduce inflammatory responses in cancer patients. Kozjek et al. found that enteral glutamine supplementation exhibited anti-inflammatory activity and reduced the hormonal stress response compared with placebo treatment in patients with rectal cancer undergoing preoperative radiochemotherapy [27]. Another double-blind, randomized, controlled pilot trial provided evidence that oral glutamine decreased the inflammatory response and abolished the changes in the autophagy machinery in patients receiving abdominal radiotherapy [28]. Glutamine supplementation has good application potential in reducing the inflammatory response and improving the immunity of tumour patients.

The results based on clinical data have shown that low serum preoperative glutamine levels are associated with a higher risk of postoperative recurrence and metastasis. An increasing number of studies have demonstrated that EMT is a key step in CRC progression and metastasis. We further analysed how glutamine depletion affects EMT and metastasis and the regulation of EMT-TFs and signalling pathways in colorectal cancer cells in this study. We found that glutamine deprivation promoted migration and invasion by reducing E-cadherin expression. In addition, glutamine deprivation induced the mRNA expression of EMT transcription factors, including zeb1, zeb2 and snail2, the protein expression of zeb1 and zeb2 were also significantly increased in glutamine deficiency groups compared with the normal glutamine group. Glutamine deficiency might reduce E-cadherin in colorectal cancer through increasing zeb1 and zeb2, further studies are needed to confirm this finding.

In human CRC tumour tissues, the glutamine concentration was significantly lower in tumours than in healthy tissues [29], and this phenomenon was also found in intestinal tumours from heterozygous APC mutant mice [30]. Glutamine depletion activates the Wnt signalling pathway and enhances cancer stemness by decreasing intracellular alpha-ketoglutarate levels; in contrast, alpha-ketoglutarate supplementation suppresses Wnt signalling and promotes cellular differentiation, thereby significantly restricting CRC growth and extending survival. However, the effect of glutamine depletion on EMT and metastasis and the underlying mechanism in CRC have not been explored. Our results were similar to those reported in pancreatic ductal adenocarcinoma (PDAC). In PDAC, glutamine was the most depleted amino acid in tumours relative to adjacent nonneoplastic tissue [31]. The same result was found in a murine PDAC model, and glutamine deprivation led to the induction of EMT in PDAC cells through the upregulation of the EMT core regulator slug [32]. A recent study demonstrated that glutamine deprivation promoted cancer-associated fibroblast migration and invasion, which in turn facilitated the invasion of tumour epithelial cells towards nutrient-rich adjacent tissues [33].

## Conclusions

In conclusion, our study demonstrated that preoperative serum glutamine is an independent prognostic biomarker to predict CRC progression. Glutamine deprivation promotes migration and invasion in CRC cells by inducing the EMT process. These results reveal that serum metabolites might play an important role in improving the current ability to predict CRC outcomes and provide mechanistic insight into how metabolic stress influences CRC progression.

## Supplementary Information

**Additional file 1: Table S1.** Primer sequences used for real-time PCR. **Table S2.** Associations of serum glutamine level with systemic inflammation of CRC patients. **Figure S1.** Associations between serum glutamine level and CEA level in CRC patients. Higher serum level of glutamine correlated with lower CEA level (*r* = − 0.25, *P* = 0.02). **Figure S2.** ROC curve of serum glutamine in predicting OS. The optimal glutamine cutoff value based on the OS was 392.83 μM, with an area under the curve of 0.646 (95% confidence interval (CI) 0.554–0.731; *P* < 0.05).

## Data Availability

The datasets used and analysed during the current study are available from the corresponding author on reasonable request.

## Abbreviations

CRC: Colorectal cancer
CEA: Carcinoembryonic antigen
OS: Overall survival
DFS: Disease-free survival
EMT: Epithelial–mesenchymal transition
SD: Standard deviation
ROC: Receiver operating characteristic
NLR: Neutrophil/lymphocyte ratio
dNLR: Derived neutrophil–lymphocyte ratio
PLR: Platelet/lymphocyte ratio
COP–NLR: The combination of platelet count and neutrophil lymphocyte
LMR: Lymphocyte/monocyte ratio
PNI: Prognostic nutritional index
PDAC: Pancreatic ductal adenocarcinoma

## References

[1] Bray F, Ferlay J, Soerjomataram I, Siegel RL, Torre LA, Jemal A (2018). Global cancer statistics 2018: GLOBOCAN estimates of incidence and mortality worldwide for 36 cancers in 185 countries. CA Cancer J Clin.

[2] Brenner H, Kloor M, Pox CP (2014). Colorectal cancer. Lancet.

[3] Cluntun AA, Lukey MJ, Cerione RA, Locasale JW (2017). Glutamine metabolism in cancer: understanding the heterogeneity. Trends Cancer.

[4] Dang CV (2010). Glutaminolysis: supplying carbon or nitrogen or both for cancer cells?. Cell Cycle.

[5] Ling HH, Pan YP, Fan CW, Tseng WK, Huang JS, Wu TH (2019). Clinical significance of serum glutamine level in patients with colorectal cancer. Nutrients.

[6] Sirnio P, Vayrynen JP, Klintrup K, Makela J, Karhu T, Herzig KH (2019). Alterations in serum amino-acid profile in the progression of colorectal cancer: associations with systemic inflammation, tumour stage and patient survival. Br J Cancer.

[7] Vu T, Datta PK (2017). Regulation of EMT in colorectal cancer: a culprit in metastasis. Cancers (Basel).

[8] Guaita S, Puig I, Franci C, Garrido M, Dominguez D, Batlle E (2002). Snail induction of epithelial to mesenchymal transition in tumor cells is accompanied by MUC1 repression and ZEB1 expression. J Biol Chem.

[9] Yang J, Mani SA, Donaher JL, Ramaswamy S, Itzykson RA, Come C (2004). Twist, a master regulator of morphogenesis, plays an essential role in tumor metastasis. Cell.

[10] Shioiri M, Shida T, Koda K, Oda K, Seike K, Nishimura M (2006). Slug expression is an independent prognostic parameter for poor survival in colorectal carcinoma patients. Br J Cancer.

[11] Kahlert C, Lahes S, Radhakrishnan P, Dutta S, Mogler C, Herpel E (2011). Overexpression of ZEB2 at the invasion front of colorectal cancer is an independent prognostic marker and regulates tumor invasion in vitro. Clin Cancer Res.

[12] Yook JI, Li XY, Ota I, Fearon ER, Weiss SJ (2005). Wnt-dependent regulation of the E-cadherin repressor snail. J Biol Chem.

[13] Voorneveld PW, Kodach LL, Jacobs RJ, Liv N, Zonnevylle AC, Hoogenboom JP (2014). Loss of SMAD4 alters BMP signaling to promote colorectal cancer cell metastasis via activation of Rho and ROCK. Gastroenterology.

[14] Hao Y, Samuels Y, Li Q, Krokowski D, Guan BJ, Wang C (2016). Oncogenic PIK3CA mutations reprogram glutamine metabolism in colorectal cancer. Nat Commun.

[15] Vander Heiden MG, Cantley LC, Thompson CB (2009). Understanding the Warburg effect: the metabolic requirements of cell proliferation. Science.

[16] Hensley CT, Wasti AT, DeBerardinis RJ (2013). Glutamine and cancer: cell biology, physiology, and clinical opportunities. J Clin Invest.

[17] Xiang L, Mou J, Shao B, Wei Y, Liang H, Takano N (2019). Glutaminase 1 expression in colorectal cancer cells is induced by hypoxia and required for tumor growth, invasion, and metastatic colonization. Cell Death Dis.

[18] Tan B, Qiu Y, Zou X, Chen T, Xie G, Cheng Y (2013). Metabonomics identifies serum metabolite markers of colorectal cancer. J Proteome Res.

[19] Zhu J, Djukovic D, Deng L, Gu H, Himmati F, Chiorean EG (2014). Colorectal cancer detection using targeted serum metabolic profiling. J Proteome Res.

[20] Bertini I, Cacciatore S, Jensen BV, Schou JV, Johansen JS, Kruhoffer M (2012). Metabolomic NMR fingerprinting to identify and predict survival of patients with metastatic colorectal cancer. Cancer Res.

[21] Li J, Li J, Wang H, Qi LW, Zhu Y, Lai M (2019). Tyrosine and glutamine-leucine are metabolic markers of early-stage colorectal cancers. Gastroenterology.

[22] Li X, Chen R, Li Z, Luo B, Geng W, Wu X (2020). Diagnostic value of combining miRNAs, CEA measurement and the FOBT in colorectal cancer screening. Cancer Manag Res.

[23] Zang R, Li Y, Jin R, Wang X, Lei Y, Che Y (2019). Enhancement of diagnostic performance in lung cancers by combining CEA and CA125 with autoantibodies detection. Oncoimmunology.

[24] Palmieri EM, Menga A, Martin-Perez R, Quinto A, Riera-Domingo C, De Tullio G (2017). Pharmacologic or genetic targeting of glutamine synthetase skews macrophages toward an M1-like phenotype and inhibits tumor metastasis. Cell Rep.

[25] Newsholme P (2001). Why is l-glutamine metabolism important to cells of the immune system in health, postinjury, surgery or infection?. J Nutr.

[26] Cruzat VF, Krause M, Newsholme P (2014). Amino acid supplementation and impact on immune function in the context of exercise. J Int Soc Sports Nutr.

[27] RotovnikKozjek N, Kompan L, Zagar T, Mrevlje Z (2017). Influence of enteral glutamine on inflammatory and hormonal response in patients with rectal cancer during preoperative radiochemotherapy. Eur J Clin Nutr.

[28] de Urbina JJO, San-Miguel B, Vidal-Casariego A, Crespo I, Sanchez DI, Mauriz JL (2017). Effects of oral glutamine on inflammatory and autophagy responses in cancer patients treated with abdominal radiotherapy: a pilot randomized trial. Int J Med Sci.

[29] Denkert C, Budczies J, Weichert W, Wohlgemuth G, Scholz M, Kind T (2008). Metabolite profiling of human colon carcinoma-deregulation of TCA cycle and amino acid turnover. Mol Cancer.

[30] Tran TQ, Hanse EA, Habowski AN, Li H, Gabra MBI, Yang Y (2020). alpha-Ketoglutarate attenuates Wnt signaling and drives differentiation in colorectal cancer. Nat Cancer.

[31] Kamphorst JJ, Nofal M, Commisso C, Hackett SR, Lu W, Grabocka E (2015). Human pancreatic cancer tumors are nutrient poor and tumor cells actively scavenge extracellular protein. Cancer Res.

[32] Recouvreux MV, Moldenhauer MR, Galenkamp KMO, Jung M, James B, Zhang Y (2020). Glutamine depletion regulates Slug to promote EMT and metastasis in pancreatic cancer. J Exp Med.

[33] Mestre-Farrera A, Bruch-Oms M, Pena R, Rodriguez-Morato J, Alba-Castellon L, Comerma L (2021). Glutamine-directed migration of cancer-activated fibroblasts facilitates epithelial tumor invasion. Cancer Res.

