# Supplementary material for: Glutamine deficiency promotes recurrence and metastasis in colorectal cancer through enhancing epithelial–mesenchymal transition
Source: J Transl Med. 2022 Jul 22;20:330. doi: 10.1186/s12967-022-03523-3 (PMC9308325; doi:10.1186/s12967-022-03523-3)
Supplement: Supplementary file 1 — Additional file 1: Table S1. Primer sequences used for real-time PCR. Table S2. Associations of serum glutamine level with systemic inflammation of CRC patients. Figure S1. Associations between serum glutamine level and CEA level in CRC patients. Higher serum level of glutamine correlated with lower CEA level (r = − 0.25, P = 0.02). Figure S2. ROC curve of serum glutamine in predicting OS. The optimal glutamine cutoff value based on the OS was 392.83 μM, with an area under the curve of 0.646 (95% confidence interval (CI) 0.554–0.731; P < 0.05). [file 12967_2022_3523_MOESM1_ESM.docx]

**Additional file 1**

Table S1 Primer sequences used for real-time PCR

| Gene | Sense (5’to 3’) | Anti-sense(5’to 3’) | Size (bp) |
| --- | --- | --- | --- |
| Snail1 | TCGGAAGCCTAACTACAGCGA | AGATGAGCATTGGCAGCGAG | 140 |
| Snail2 | GCCAAACTACAGCGAACTGG | GATGGGGCTGTATGCTCCTG | 121 |
| Zeb1 | GATGATGAATGCGAGTCAGATGC | ACAGCAGTGTCTTGTTGTTGT | 86 |
| Zeb2 | GGAGACGAGTCCAGCTAGTGT | CCACTCCACCCTCCCTTATTTC | 107 |
| Gapdh | AGAAGGCTGGGGCTCATTTG | AGGGGCCATCCACAGTCTTC | 258 |

Table S2 Associations of serum glutamine level with systemic inflammation of CRC patients

| Variable | No. of cases | Glutamine (μM) | | *P* values |
| --- | --- | --- | --- | --- |
|  |  | mean | SD |  |
| **NLR** |  |  |  | 0.478 |
| ≥5 | 31 | 581.45 | 327.25 |  |
| <5 | 90 | 525.18 | 395.79 |  |
| **dNLR** |  |  |  | 0.537 |
| ≥3 | 32 | 575.26 | 326.10 |  |
| <3 | 89 | 526.77 | 397.08 |  |
| **PLR** |  |  |  | 0.346 |
| >300 | 19 | 516.73 | 276.05 |  |
| 150–300 | 46 | 603.23 | 416.62 |  |
| <150 | 56 | 495.08 | 375.09 |  |
| **COP–NLR** |  |  |  | 0.810 |
| NLR>3 and platelet count >300*10^9^/L(Score 2) | 18 | 580.96 | 412.97 |  |
| NLR>3 or platelet count >300*10^9^/L(Score 1) | 50 | 516.28 | 385.06 |  |
| Nether NLR>3 nor platelet count >300*10^9^/L(Score 0) | 53 | 547.55 | 366.93 |  |
| **LMR** |  |  |  | 0.391 |
| ≥2.35 | 83 | 519.51 | 393.68 |  |
| <2.35 | 38 | 583.46 | 345.31 |  |
| **PNI** |  |  |  | 0.631 |
| ≥45 | 68 | 524.91 | 367.15 |  |
| <45 | 53 | 558.44 | 396.19 |  |

NLR, neutrophil/lymphocyte ratio; dNLR, derived neutrophil–lymphocyte ratio; PLR, platelet/lymphocyte ratio ; COP–NLR, the combination of platelet count and neutrophil lymphocyte LMR, lymphocyte/monocyte ratio ;PNI, prognostic nutritional index

**
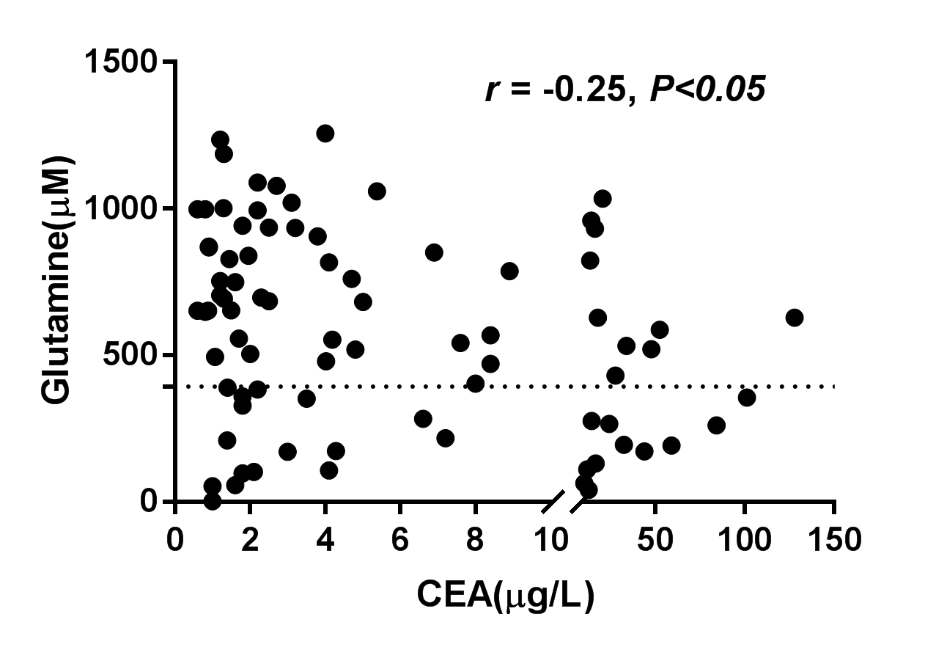
**

Fig. S1 Associations between serum glutamine level and CEA level in CRC patients. Higher serum level of glutamine correlated with lower CEA level (*r*=-0.25, *P*=0.02).

**
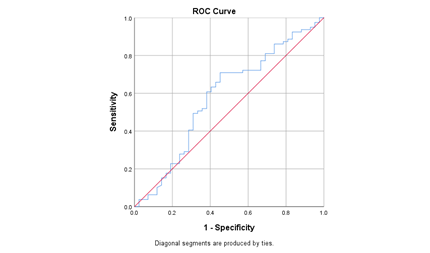
**

Fig. S2 ROC curve of serum glutamine in predicting OS. The optimal glutamine cutoff value based on the OS was 392.83μM, with an area under the curve of 0.646 (95% confidence interval (CI): 0.554–0.731; *P* < 0.05)
